# Children’s early helping in action: Piagetian developmental theory and early prosocial behavior

**DOI:** 10.3389/fpsyg.2014.00759

**Published:** 2014-07-17

**Authors:** Stuart I. Hammond

**Affiliations:** School of Psychology, University of OttawaOttawa, ON, Canada

**Keywords:** prosocial behavior, moral development, social interaction

## Abstract

After a brief overview of recent research on early helping, outlining some central problems, and issues, this paper examines children’s early helping through the lens of Piagetian moral and developmental theory, drawing on Piaget’s “Moral Judgment of the Child” (Piaget, 1932/1997), “Play, Dreams, and Imitation in Childhood” (Piaget, 1945/1951), and the “Grasp of Consciousness” (Piaget, 1976). Piaget refers to a level of moral development in action that precedes heteronomous and autonomous moral reasoning. This action level allows children to begin to interact with people and objects. In his later work, Piaget explores the gradual construction of understanding from this activity level. Taken together, these elements of Piagetian theory provide a promising conceptual framework for understanding the development of early helping.

Young children begin to help others soon after their first birthday (Warneken and Tomasello, 2007). In the lab, toddlers will assist adults who have encountered a variety of problems, with a progression of helping behaviors. Children’s earliest form of helping, appearing as early as 14 months (Warneken and Tomasello, 2007), is often called *instrumental helping*, as it involves aiding an adult complete a thwarted goal, such as retrieving a dropped object, or opening a door (e.g., Warneken and Tomasello, 2006; Svetlova et al., 2010; Dunfield et al., 2011). Early in the second year of life, children begin to display *empathic* helping, which is oriented at relieving those in distress, such as providing a blanket to someone who is cold. Closer to 30 months of age, children also begin to show *altruistic* helping, which involves sharing and distributing resources, including those belonging to the child (e.g., loaning a favorite toy). In many traditional models of prosocial behavior, these forms of helping would be attributed to skills such as emotion understanding and perspective taking. Yet developmentally, the appearance of early helping precedes these forms of complex social emotional understanding, leaving the emergence of help, its developmental trends, and sources of individual differences poorly understood (Svetlova et al., 2010; Paulus, 2014).

Doubtlessly, some of the difficulties in understanding early help are conceptual. Help is a term of natural language, not a technical or psychological term, and does not have a precise psychological correlate. Helping can involve many different actions and situations, ranging from doing something explicitly requested or commanded (e.g., “Can you hand me that hammer?”; “Come here!”) to doing something that we feel is another’s interest (e.g., opening a door for someone coming into a building behind us; assisting someone who is hurt). As Paulus (2014) remarks, helping in these situations could involve a plethora of psychological skills, despite the superficial unity implied by the word “help.” There is also a moral connotation to the term help, which again may or may not apply to the particulars of a situation. In the lab, helping opportunities are generally structured such that children’s participation is prosocial. But in real life, assisting another achieve a goal (e.g., retrieving car keys for someone who is drunk) is not always the right thing to do. Furthermore, help can be in the eye of the beholder; for example, a parent’s decision to prevent their child from playing video games, even if aimed at assisting the child achieve in school, may go unappreciated by the child. The issue of perspective may be important to the development of helping, as young children’s assistance in routines and chores in the home, although often unhelpful to parents, slowing down and stymying their efforts, is also regarded by many parents as worthy of encouraging and supporting (Rheingold, 1982; Hammond and Brownell, 2014).

Despite the unresolved complexities that surround the concept of help, recent studies on early helping have many raised questions about human evolution, development, and morality. For some, the early appearance of helping could suggest – in the sense of behavioral ontogeny recapitulating behavioral phylogeny – that human cooperativeness emerged at an earlier evolutionary time (Warneken and Tomasello, 2009). This would suggest that a predominant view of human nature as selfish is misguided (Hay, 2009). The precocity of helping also raises questions about how it is learned. Although socialization seems like a plausible route, Warneken and Tomasello (2013) found that instrumental helping is not influenced by social praise and reinforcement. Furthermore, they argue that because young children are unlikely to encounter situations where an adult needs their help in the home, they have few opportunities to learn about helping. Instead, it seems that “very young children have a natural tendency to help other persons solve their problems” (Warneken and Tomasello, 2006, p. 1302). With evolutionary roots and early, unexplained, appearance, helping seems to be a good candidate for the larger movement in developmental psychology that posits infants possess some elements of an innate morality (e.g., Hamlin et al., 2007; Bloom, 2012).

However, Warneken and Tomasello’s (2006) argument relies on a relatively narrow view of learning, one that omits situations in which children apply skills learned in one context to other contexts. Hebb et al. (1971) call this latter form of learned behavior, behavior “that is not learned, but *is* dependent on prior learning” (p. 213). Although no one has systematically investigated the frequency with which adults encounter problems that require a child’s aid, children clearly can and do get involved in helping their parents in the home (Rheingold, 1982; Hay, 2009). The social ecology of the home reveals a rich context in which children could develop skills that may allow them to offer instrumental help, i.e., helping in a situation where an adult encounters a problem, even if they have never encountered this particular situation outside the lab.

Unfortunately for parents, and in contrast with the view that children come into the world biologically prepared to help others, young children’s “help” is often less than helpful. One- and 2-year-olds’ involvement in activities in the home often involves interfering with whatever their parents are doing, smearing spills instead of wiping them up, or setting clean dishes from the dishwasher on the floor. Many young children insist that they be allowed to assist with these activities, and may grow angry if they are excluded (Forman, 2007). Children’s “uncooperative cooperativeness” leads many parents to wait until their children are napping to take care of household tasks (Rheingold, 1982). But, parents also report that they want to have their children take part in this help, as, in the words of one mother, “it is usually unhelpful and makes everything take longer, but she loves learning new things and it is my job as mommy to teach her all of these things” (Hammond and Brownell, 2014). Eventually, children do learn to help in the home. As diary and ethnographic studies show, young children can come to play important, and genuinely helpful, roles in the home and larger community (e.g., Rogoff, 2003; Hay, 2009). However, children also come lose their zeal for some of these tasks, and must be coaxed to do chores and help their parents, suggesting developmental changes in motivation.

How can these diverse pictures of children’s early helping, of children as natural altruists and unhelpful helpers, be reconciled? At this juncture in research on early helping, there may be value in revisiting an older theory of moral development for a better understanding of early helping. The recent trend in developmental research towards innate morality is in some ways a pushback against the seminal work of Piaget (1932/1997), whose explorations of morality are largely restricted to later childhood (e.g., Hamlin, 2013). The reaction against Piaget’s influence, which has occurred in many other areas of developmental psychology (e.g., object knowledge; numerical cognition; social cognition, etc.), has had the positive effect of demonstrating that many phenomena appear earlier in the lifespan than expected (Hay, 2009). However, these efforts to frame early helping as unlearned, and infant morality as largely equivalent to its adult forms, risk missing out on the unique developmental features of early helping, which are particularly evident in observations of children in the home, and the developmental trends in helping such as the transition from instrumental to empathic and altruistic helping.

Although early helping clearly precedes the period of morality that Piaget was particularly interested in, the broad features of the Piagetian developmental program, which focuses on change and transformation, may be able to make some positive contributions to the study of early helping, highlighting features of early helping that have been overlooked and presenting some revised expectations about its emergence and subsequent development. In particular, Piaget’s theory, which is at its heart an account of how knowledge emerges from action (Chapman, 1988), may have something to say about how children manage to begin to help others before they have develop complex social cognitive representations. The present paper will draw on a few elements Piaget’s works, namely his early works the *Moral Judgment of the Child* (Piaget, 1932/1997) and *Play, Dreams, and Imitation in Childhood* (Piaget, 1945/1951), and his later work on the transition from action to reflective cognition, the *Grasp of Consciousness* (Piaget, 1976), to explore the emergence of early helping, the transition to more complex forms of helping, and what may happen to helping in later development.

## BEFORE MORALITY REASONING: THE EMERGENCE OF EARLY HELPING

At first glance, Piaget’s moral developmental theory, which is largely focused on children aged 5 to 12, has little to contribute to the study of early helping, which occurs in the first few years of life. Furthermore, in the opening line of the *Moral Judgment of the Child*, Piaget (1932/1997) declares that he offers, “no direct analysis of child morality as it is practiced in home and school life” (p. 10). Instead, his moral theory unfolds as a critique of the prominent moral education theory of sociologist Émile Durkheim (Durkheim, 1961; Fedi, 2008). Whereas Durkheim saw morality and moral rules thrust on the child by adult society, Piaget countered that this was only one part of the story. Adults’ unilateral exertion of rules on the child resulted in the child developing a *heteronomous* understanding of morality, where moral rules and norms were unchangeable and externally regulated through punishment. Piaget argued that children were also exposed to a different societal structure through social interactions with peers, characterized by a rough equivalence of power, and which lead to the development of an *autonomous* understanding of morality, where moral rules and norms could be constructed and negotiated in social interaction through a coordination of perspectives (Carpendale, 2009). Interestingly, despite their disagreements, both Durkheim (1888/1970) and Piaget found the idea that humans were born with innate knowledge deeply implausible.

Piaget (1932/1997) used the example of children learning the game of marbles, a social game with manifold rules (and a widespread popularity in his day and age) as a proxy for learning moral rules. As children develop, they begin to understand that the rules of marbles and morals are not unchangeable, handed down from generation to generation, but are living and breathing systems used to coordinate social interaction with others, and can be negotiated and changed. But, before children learn to think and reason about the rules of marbles, they must actually learn how to play marbles. And analogously, before children can learn to think about morals, they must actually engage in morally relevant activity. Piaget acknowledges from the outset that the practice and consciousness of morality is different, and, importantly, that the former emerges before the latter (p. 14). Even though the bulk of Piaget’s system of moral development is focused on moral reasoning, it is rooted in the practice of moral activity (Carpendale, 2009).

Piaget’s recognition that there is moral practice before complex moral representations transforms one of the developmental mysteries of children’s early helping. As Svetlova et al. (2010) remark, the central problem of early helping is how children manage to engage in it before they have developed advanced forms of social cognition and social understanding. But in a Piagetian account, this is precisely what we would expect to happen: young children should begin to learn some aspects of helping before the appearance of complex mental representations. For Piaget, moral development begins in a “stage of a purely *motor* and *individual* character… [leading to] the formation of more or less ritualized schemas” (Piaget, 1932/1997, p. 26). The child is learning to interact with objects, and people, in the world, and organize this action.

It should be noted that Piaget uses the term individual, and social, in a somewhat idiosyncratic way (Chapman, 1988). Piaget’s point is that the child’s early moral interactions are not entirely social, or not social in the same way that older children are social (Carpendale, 2009). Piaget (1932/1997) observes the child operating “in an individualistic manner with material that is social” (p. 37). By this he means that children’s interactions often reflect their own interests, rather than reflecting the goal of the other. Although children are using the same objects as others (e.g., marbles), and interacting with others to some extent, in other ways their interactions are quite different than mature forms. Children are learning to engage in some aspects of the regularity of organized practices, but they do not understand these practices’ larger goals. We could expect, transposing the theory to early helping that children may develop skills such as cleaning with others without a fully developed understanding of why these tasks are being accomplished, and of course with far more limited motor skills.

As children master the basic motor aspects of activities, they possess only a limited understanding of the greater context of these activities. As Piaget (1932/1997) puts it, these early interactions “may be called *egocentric*... the child imitates... without trying to win [i.e., the game of marbles]” (p. 27). Transposing the point about winning a game to the context of early helping, the child can be involved in activities without a good knowledge of their greater goal (e.g., play with marbles without understanding what it is to win a game of marbles; wipe a table with a cloth without understanding the goal of cleaning up the house). This fits with parental reports that children’s helping is not always helpful (Rheingold, 1982). The child is capable of participating in some aspects of the task alongside their parents, but they do not necessary have a full understanding of the goals of the task at hand.

In fact the child’s own version of the task may be quite eccentric. As one parent describes the process of doing laundry with her child, “[w]hen helping to fold laundry she often just wads it up into little balls. I try and show her the right way which lasts all of three items before she’s back to wadding them up again” (Hammond and Brownell, 2014). For a young child, collaborative helping opportunities, like putting away laundry, is a sort of game, one in which they may fixate on particularly enjoyable aspects, to the detriment of the task as a whole. The child is clearly able to take part in most aspect of the task at hand in isolation (e.g., move dirty laundry into the washing machine). But systematically, their understanding of how actions should be coordinated within the task is such that their help is unlikely to be helpful, without heavy management from parents.

Children’s unconventional manner of helping is rarely remarked upon in lab-based studies of children’s early helping. This may be because helping tasks in the lab are structured to rely largely on isolated components of helping, in such a way in that they artificially make the child seem more competent than they really are (Carpendale et al., 2013). In these studies, children are required to carry out relatively modest acts, e.g., pick up a dropped object and return it to someone’s hand. This task is explicitly structured so that if the said act is performed, it is helpful to the adult.

In the motor and individualistic aspects of early moral development, Piaget (1932/1997) also alights on some unique developmental aspects of early moral development, which is the joy children express as they come to engage in regular and ritualized interactions (p. 33). Rheingold (1982) describes children’s early helping as marked by “alacrity,” engaging in “quick and energetic movements, excited vocal intonations, animated facial expressions, and with delight in the finished task” (p. 119). Again, this feature of children’s early helping, though present in the subjects of contemporary lab-based studies of children’s early helping, are rarely remarked upon as an important feature of help in these studies, which have largely been concerned with the outcome of help (e.g., its presence and rapidity), and much less interested in its process and character.

The recognition of children’s joy in helping stands in contrast to a great deal of work in moral psychology and moral philosophy that has emphasized that helping uniquely emerges from concern and sadness (Wispé, 1991). As Adam Smith’s friend and fellow philosopher David Hume pointed out long ago in a letter to Smith, if shared misery is the basis of our moral interactions with others, then morality would play a wholly onerous role in human existence (Ross, 1995, p. 179). Developmentally, it is unclear how morality would develop if children could learn about it only in situations of pain and distress. Extending Warneken and Tomasello’s (2006) argument, these distressing situations are probably relatively rare in the child’s life to form a basis for learning to help. Children have far more experience helping others in collaborative and playful contexts, with shared joy and enjoyment (Brownell et al., 2002).

## FROM INSTRUMENTAL HELP TO EMPATHIC UNDERSTANDING: IMITATION AND SYMBOLIC UNDERSTANDING

The picture presented by Piaget’s work on early morality is that of a child learning to engage collaboratively with others, without a full understanding of others’ goals. In the rest of the *Moral Judgment*, Piaget largely leaves the early action level behind, turning instead to how children understand rules in relations of heteronomy and autonomy, an issue that lies beyond early helping, and will be returned to briefly below. However, we can turn elsewhere in the corpus of his work to learn more about how early helping might develop as children begin to reflect on helping.

In *Play, Dreams, and Imitation in Childhood* (Piaget, 1945/1951), Piaget provides many examples of children’s early activity in the context of exploratory play. The imitation of people has been accorded a great deal of importance in both recent psychological literature, such as on mirror neurons (e.g., Keysers, 2009), and early learning (Paulus et al., 2011), as well as in earlier sociological literature (e.g., Tarde, 1903). Piaget finds many examples of children imitating others’ actions and activities, such as modeling another person crossing their arms, or stamping their feet. However, perhaps setting his work apart from the focus on imitating other humans’ actions, Piaget’s work on imitation acknowledges that children imitate both people and objects. For example, Piaget (1945/1951) observes a 1-year-old child imitating “the sound of a rattling window and sway[ing] to the same rhythm” (p. 66).

As such, Piaget is not referring to imitation as merely a type of copying or mirroring, as a child cannot truly produce a copy of a rattling window as they can a walking adult, but rather as a form of learning by which they can integrate aspects of rattling window into their own repertoire of action. As children develop, they can produce these imitations, with greater ease, and manipulating them, reversing them, applying them in new situations and so on. As children manipulate these action schemes, they can accomplish a wide variety of tasks based on sensorimotor knowledge (i.e., practical knowledge) alone. This class of tasks would undoubtedly include helping others in many instrumental contexts. Young children, by the age at which helping first appears, have uncontestably experienced many of the components of helping others, whether handing over objects, or opening doors, throughout their lives. Indeed, and although Piaget badly underplayed the role of observational learning in his action theory, they have been experiencing and observing others handing objects to them for even longer. Furthermore, in the context of his larger theory of sensorimotor development, Piaget would clearly expect children to be able to apply action schema (e.g., handing an object to someone) in new contexts and novel situations (e.g., after someone has dropped it).

Piaget’s expectation that children have the ability to apply action schema to new contexts is relevant to Warneken and Tomasello’s (2006) argument that instrumental helping is likely unlearned because problem contexts are so rare in the young child’s life. As noted early, the helping paradigms in the lab focus on problem situations where an adult feigns incapacitation (e.g., that they are unable to reach a dropped object), which the child has an opportunity to resolve. These types of situations may indeed be rare in children’s lives, and when children encounter these situations in the lab, their help seems to come from nowhere. But according to Piaget, just because certain situations are rare, or novel, for the life of the child, does not necessarily mean that the child has no pertinent learning to apply in this rare or novel context. Echoing Hebb et al. (1971) view, Piaget would likely argue that children’s instrumental helping is dependent on prior learning. A great deal of the earliest instrumental helping, which involves returning dropped and out-of-reach objects to the hands of an adult (e.g., Warneken and Tomasello, 2007), could in principle be explained by this ability to quickly emulate, and reverse, the movement of people and of the objects. The act of returning something to someone’s hand is something the child has likely seen, and experienced themselves (e.g., when they have dropped their bowl of food), many times, and is not particularly difficult for a young child to do.

That said, children encounter situations in which their prior learning cannot aid them. A likely candidate seems to be empathic helping, which appears later in the lifespan than instrumental helping. Empathic helping occurs in situations such as someone shivering with cold, and who needs a blanket (e.g., Svetlova et al., 2010). In this type of scenario, young children clearly have the requisite skills to lift and carry a blanket over to a shivering adult. But how do they learn to connect the blanket to a person shivering? This helping situation is structured radically differently than instrumental helping tasks. When a person is shivering, there is no interaction between person and object. Instead the interaction is situated only with the experimenter in their shivering. Shivering can be symbolically linked to needing a blanket, but there is no direct interaction with this blanket. To solve the empathic task at a stage where no interaction has taken place, the child must recognize the meaning of someone shivering and its relation to a blanket somewhere else in the room.

In experimental studies, should the child fail to initially retrieve the blanket, the experimenter subsequently begins to reach for the blanket. It is here that an imitatable interaction emerges, and this also seems to be the point when younger children begin to retrieve the blanket (Svetlova et al., 2010). Thus, the empathic forms of helping may emerge later for children, not because they rely on “hidden” emotions or mental states (after all, shivering is an overt behavior), but because the emotional displays involved do not initially include a direct interaction between the experimenter and object needed to solve the task, but rely on a more symbolic form of understanding (“a blanket is for a person who is cold”; “a person who is shivering is cold”). This relation between shivering and a blanket is not apparent in the undifferentiated action context, because there is no interaction between the person and the blanket. Although it is possible “solve” empathic helping tasks with sensorimotor skills, a far more elegant solution lies in the child learning to understand the world more symbolically, explicitly bringing actions (e.g., shivering) into relation with objects (e.g., a blanket).

## UNDERSTANDING HELPING OTHERS: THE GRASP OF CONSCIOUSNESS

Piaget’s work suggests that children can learn to help with sensory and motor skills. However, children eventually learn to differentiate the world into self and other, object and person, and gain more mentalistic and reflective skills. In the *Grasp of Consciousness*, Piaget (1976) lays out that the process by which children gain reflective understanding, as their practical knowledge is reconstructed on a conscious plane, or level, of thought. This conscious reconstruction isn’t merely an “illumination” of what was occurring on the plane of action, i.e., a direct isomorphism of that activity, but a reconstruction that moves beyond what they see in direct interaction, and thereby allows them to reorganize their existing activity in more sophisticated ways (Campbell and Bickhard, 1986).

In Piaget’s account, reflective understanding arises first in what Piaget (1976) calls the *periphery*, and only gradually moving to what he calls the *center*. In this somewhat misleading terminology, the periphery refers to the site of interaction between subject and object, whereas the center(s) refers to characteristics associated with the subject(s) and object(s) involved in the interaction. Piaget assigns the terms periphery and center on the basis of their importance to explaining causality, i.e., the characteristics of subject and object are centers in terms of a causal explanation of an interaction, where the interaction itself only offers some peripheral aspects. Although Piaget lays out the periphery-center model in the context of the child-as-subject interacting with some object, there seems to be no reason why cognizance could not also involve other subjects as other centers.

In this periphery-center model, children’s understanding will first form around aspects of interaction between object and subject, and only later begin to form around the properties of objects and subjects contribute to these interactions. So for example, early on, a child may understand that opening a door will allow toys to be retrieved from a closet (an interaction), but the same child is unlikely to understand the properties of the mechanisms of the door (central characteristics of the object), nor successfully reflect on how they learned to open the door (central characteristics of the subject).

In that children focus on the periphery, i.e., the point of interaction, Piaget’s work suggests that in the early stages of development, children begin to understand objects and people in a relatively undifferentiated way. When a child sees someone drop a pen they likely approach the situation holistically, understand something about people and pens, i.e., that pens belong in a hand, rather than segmenting the situation into one in which they must read the mind of the other. The child’s knowledge begins with interactions between people and objects (e.g., a marker falling out of someone’s hand). These are only later constructed into differentiated knowledge about objects (e.g., blankets can make people warm) and subjects (e.g., shivering means cold) proper. The fact that early practical knowledge blends subject and object in interaction means that children do not *a priori* distinguish people/minds as one form of knowledge, and objects and things as another (Bibok et al., 2008). However, research on children’s early helping, like so much of psychology, has fixated on the problem of other minds, such that one part of the reason we are surprised by the precocity of children’s early helping is that we presuppose that this helping must involve a knowledge of other minds, rather than framing the issue as skills in interaction.

If this principle of periphery to center is applied to the context of helping, children would be expected to first understand aspects of interaction, such as retrieving a dropped object and placing it in the hand of an experimenter, long before they understand the central social cognitive questions of why the experimenter was unable to retrieve the dropped object. Nearly all lab-based instrumental helping tasks are structured with peripheral components, such as an experimenter with an arm full of books bumping up against a door he or she cannot open. Only later will children be able to differentiate and reflect on the more central aspects of helping, such as the larger goals and capacities and needs of the helpee.

One recent study of helping in slightly older children shows that with development, children’s helping seems to become more attuned to discriminate when someone actually needs help versus someone who is capable of solving the problem on their own (Paulus and Moore, 2011). In the home, this restriction to the peripheral may explain why children can initially carry out many of the components of helping, e.g., picking up laundry, even if they do not understand their parents’ larger goals, nor quite see some important characteristics of the objects involved (e.g., dirty vs. clean clothes). As children develop, they may learn to reflect on these more central aspects. Interestingly, and in what is an important point of future longitudinal investigation, this may mean that children help less as they get older, even as they become more competent helpers.

## DIFFERENTIATING HELP: FUTURE DIRECTIONS OF CHILDREN’S EARLY HELPING

The literature on children’s early helping has thus far largely focused on the presence of help, rather than individual differences. In reviewing Piaget’s moral theory, an important point to address, and correct, is the role that Piaget sees for parent–child social interaction in moral development, and how this might be a source of these differences. Piaget is somewhat infamous for emphasizing the deleterious role of parent in moral development. However, Piaget’s characterization of adult society as being detrimental to the development morality was in fact an emphasis on an ideal type of authoritarian parenting (Vidal, 1998). Piaget did not programmatically view parental involvement as necessarily injurious to the children’s moral development (Carpendale, 2009).

A more serious lacunae in Piaget’s model of social development was his tendency to generally downplay the content of early interactions, and the way in which these expose children to skills in a variety of social and cultural contexts (e.g., caring for children; preparing food). Instead, Piaget largely focused on the power structure of these interactions, in so far as these promoted obligation or mutual respect (Moessinger, 2008). These structural issues may be important in terms of the quality of parent-child interactions, such as scaffolding, which may provide a route to explaining individual differences in children’s helping (Pettygrove et al., 2013). Nevertheless, Piaget’s writing on early moral development leaves room for profound parental influences on children’s early helping activities, as parents socialize and introduce the child to a variety of different practices (Grusec et al., 2013). Parents, and social institutions, provide opportunities for children to learn a wide variety of skills. As Rogoff (2003) remarks, in some cultures, young children are trained in skills, such as using machetes to cut food, that but seem almost mind-boggling dangerous in Western society.

Beyond the issue of the types helping children become proficient in, there is the issue of the connection between this early form of moral behavior and later moral development. In Piaget’s theory, aspects of children’s early helping behavior will become conceptualized by the child and become part of the child’s moral understanding. Here we may expect further individual differences. The joy that children display in early helping, their enthusiasm and insistence to get involved in the tasks of adults, changes in structure and motivation in early and middle childhood. Following Piaget’s theory of moral development, children may come to conceptualize certain forms of helping heteronomously and imposed by obligation. So a chore, such as vacuuming, which was interesting when the child was delighted in the action of pushing the vacuum might becomes something they feel forced to do. But children may come to understand other forms of helping differently, particularly if these are negotiated in a relation of respect and caring for the other. An example of this sort of helping might be feeding the family pet.

## CONCLUSION

In this brief first pass, Piaget’s theory offers some interesting avenues to rethink some of the major problems facing research on children’s early helping. In the Piagetian account, children should be expected to begin to help before the formation of complex mental representations (Svetlova et al., 2010). However, this early helping will also be characterized by properties such as enthusiasm and unhelpful helping that we would not expect of its mature forms (Rheingold, 1982). Piagetian theory also suggests a way to resolve Warneken and Tomasello’s (2006) view that children’s help in problem situations cannot be learned because these situations are so rare in the child’s life. The Piagetian concept of learning would allow children’s skills learned in one context to transfer to novel contexts (e.g., a problem scenario).

Piaget’s action theory suggests some developmental expectations for why certain forms of helping are more difficult for children than others. Early on, children are best able to understand interactions (e.g., someone reaching for a dropped marker), only later do they begin to be able to understand more disassociated aspects of helping (e.g., someone shivering with cold). The types of practical activities the child is exposed to by both their parent and culture may form the basis of their early helping. Finally, Piaget’s theory suggests how children might come to understand their help in different ways, and with different consequences, depending on whether they view this help in the context of obligation or mutual respect.

In her own seminal work on early helping, Rheingold (1982) wondered whether the “attribution of the terms ‘sharing,’ ‘comforting,’ or ‘helping’ to very young children may appear unjustified to those who wish to reserve the terms for persons old enough to verbalize their intentions and... be explicitly aware of their motives” (p. 114). As he did with other developmental phenomena, Piaget would likely give a developmental answer, rather than a clear-cut yes or no (e.g., Piaget and Inhelder, 1966) and suggest that children’s early helping may be only the first step of a much longer journey.

## Conflict of Interest Statement

The author declares that the research was conducted in the absence of any commercial or financial relationships that could be construed as a potential conflict of interest.

## References

[B1] Bibok M. B. Carpendale J. I. M. Lewis C. (2008). “Social knowledge as social skill,” in *Social Life and Social Knowledge* eds Müller U. Carpendale J. I. M. Budwig N. Sokol B. W. (New York, NY: Lawrence Erlbaum Associates) 145–169

[B2] Bloom P. (2012). “Moral nativism and moral psychology,” in *The Social Psychology of Morality: Exploring the Causes of Good and Evil* eds Mikulincer M. Shaver P. R. (Washington, DC: APA) 71–89

[B3] Brownell C. A. Zerwas S. Balaram G. (2002). Peers, cooperative play, and the development of empathy in children. *Behav. Brain Sci.* 25 28–29 10.1017/S0140525X02300013

[B4] Campbell R. L. Bickhard M. H. (1986). *Knowing Levels and Developmental Stages.* New York: Karger

[B5] Carpendale J. I. M. (2009). “Piaget’s theory of moral development,” in *Cambridge Companion to Piaget* eds Müller U. Carpendale J. I. M. Smith L. (New York: Cambridge University Press) 270–286 10.1017/CCOL9780521898584.012

[B6] Carpendale J. I. M. Hammond S. I. Atwood S. (2013). “A relational developmental systems approach to moral development,” in *Advances in Child Development and Behavior* Vol. 45 eds Lerner R. M. Benson J. B. (Amsterdam: Elsevier Science) 123–153 10.1016/b978-0-12-397946-9.00006-3 23865115

[B7] Chapman M. (1988). *Constructive Evolution: Origins and Development of Piaget’s Thought*. New York, NY: Cambridge University Press

[B8] Dunfield K. Kuhlmeier V. A. O’Connell L. Kelley E. (2011). Examining the diversity of prosocial behavior. *Infancy* 16 227–247 10.1111/j.1532-7078.2010.00041.x 32693496

[B9] Durkheim É. (1961). *Moral Education.* New York: The Free Press (Original work published 1925)

[B10] Durkheim É. (1888/1970). “Cours de science sociale,” in *La Science Sociale et l’action* ed. Filloux J.-C. (Paris: Presses Universitaires de France) 77–110

[B11] Fedi L. (2008). *Piaget et la Conscience Morale.* Paris: Presses Universitaires de France

[B12] Forman D. R. (2007). “Autonomy, compliance, and internalization,” in *Socioemotional Development in the Toddler Years: Transitions and Transformations* eds Brownell C. A. Kopp C. B. (New York, NY: Guilford Press) 285–319

[B13] Grusec J. E. Chaparro M. P. Johnston M. Sherman A. (2013). “The development of moral behaviour and conscience from a socialization perspective,” in *Handbook of Moral Development* 2nd Edn eds Killen M. Smetana J. G. (New York: Psychology Press) 113–134

[B14] Hamlin J. K. (2013). Failed attempts to help and harm: intention versus outcome in preverbal infants’ social evaluations. *Cognition* 128 451–474 10.1016/j.cognition.2013.04.004 23811094

[B15] Hamlin J. K. Wynn K. Bloom P. (2007). Social evaluation by preverbal infants. *Nature* 450 557–559 10.1038/nature06288 18033298

[B16] Hammond S. I. Brownell C. A. (2014). “What makes mommy and daddy’s little helper tick? Motivational factors involved in prosociality in the home,” in *Poster presented at the Development 2014* Ottawa, ON

[B17] Hay D. F. (2009). The roots and branches of human altruism. *Br. J. Psychol.* 100 473–479 10.1348/000712609X442096 19467174

[B18] Hebb D. O. Lambert W. E. Tucker G. R. (1971). Language, thought and experience. *Mod. Lang. J.* 55 212–222

[B19] Keysers C. (2009). Mirror neurons. *Curr. Biol.* 19 R971–R973 10.1016/j.cub.2009.08.026 19922849

[B20] Moessinger P. (2008). *Voir la Société*. Paris: Hermann Éditeurs

[B21] Paulus M. (2014). The emergence of prosocial behavior: why do infants and toddlers help, comfort, and share? *Child Dev. Perspect.* 8 77–81 10.1111/cdep.12066

[B22] Paulus M. Hunnius S. Vissers M. Bekkering H. (2011). Imitation in infancy: rational or motor resonance? *Child Dev.* 82 1047–1057 10.1111/j.1467-8624.2011.01610.x 21679175

[B23] Paulus M. Moore C. (2011). Whom to ask for help? *Exp. Brain Res.* 211 593–600 10.1007/s00221-011-2676-1 21509491 PMC3102193

[B24] Pettygrove D. M. Hammond S. I. Karahuta E. L. Waugh W. E. Brownell C. A. (2013). From cleaning up to helping out: parental socialization and children’s early prosocial behavior. *Infant Behav. Dev.* 36 843–846 10.1016/j.infbeh.2013.09.005 24140842

[B25] Piaget J. (1951). *Play, Dreams, and Imitation in Childhood*. London: W. Heinemann (Original work published 1945)

[B26] Piaget J. (1976). *The Grasp of Consciousness.* Cambridge, MA: Harvard University Press

[B27] Piaget J. (1997). *The Moral Judgment of the Child.* New York: The Free Press (Original work published 1932)

[B28] Piaget J. Inhelder B. (1966). *La Psychologie de l’enfant.* Vendôme: Presses Universitaires de France

[B29] Rheingold H. L. (1982). Little children’s participation in the work of adults, a nascent prosocial behavior. *Child Dev.* 53 114–125 10.2307/1129643

[B30] Rogoff B. (2003). *The Cultural Nature of Human Development.* New York: Oxford University Press

[B31] Ross I. S. (1995). *The Life of Adam Smith.* New York: Oxford University Press 10.1093/0198288212.001.0001

[B32] Svetlova M. Nichols S. R. Brownell C. A. (2010). From instrumental to empathic to altruistic helping. *Child Dev.* 81 1814–1827 10.1111/j.1467-8624.2010.01512.x 21077866 PMC3088085

[B33] Tarde G. D. (1903). *The Laws of Imitation.* New York: Henry Holt and Company

[B34] Vidal F. (1998). Immanence, affectivité et démocratie dans Le jugement moral chez l’enfant. *Bull. Psychol.* 51 585–597

[B35] Warneken F. Tomasello M. (2006). Altruistic helping in human infants and young chimpanzees. *Science* 311 1301–1303 10.1126/science.1121448 16513986

[B36] Warneken F. Tomasello M. (2007). Helping and cooperation at 14 months of age. *Infancy* 11 271–294 10.1111/j.1532-7078.2007.tb00227.x 33412734

[B37] Warneken F. Tomasello M. (2009). The roots of human altruism. *Br. J. Psychol.* 100 455–471 10.1348/000712608X379061 19063815

[B38] Warneken F. Tomasello M. (2013). Parental presence and encouragement do not influence helping in young children. *Infancy* 18 345–368 10.1111/j.1532-7078.2012.00120.x

[B39] Wispé L. (1991). *The Psychology of Sympathy.* New York: Plenum Press 10.1007/978-1-4757-6779-7

